# The Clinical Characteristics, Treatment, and Prognosis of Lung Cancer in Young Patients in the New Era of Cancer Treatment: A Retrospective and Comprehensive Analysis

**DOI:** 10.3390/curroncol32090489

**Published:** 2025-08-31

**Authors:** Xiaoyi Feng, Shengjie Li, Siyuan Yu, Yunxin Liu, Zhanxian Peng, Haoran Zhang, Xiaoxing Gao, Xiaoyan Liu, Minjiang Chen, Jing Zhao, Wei Zhong, Yan Xu, Mengzhao Wang

**Affiliations:** 1 Department of Respiratory and Critical Care Medicine, Peking Union Medical College Hospital, Chinese Academy of Medical Sciences and Peking Union Medical College, Beijing 100730, China; b2023001140@pumc.edu.cn (X.F.); yusiyuan@pumc.edu.cn (S.Y.); yunxin.liu@pumc.edu.cn (Y.L.); b2021001089@pumc.edu.cn (H.Z.); gaoxiaoxing@pumch.cn (X.G.); liuxiaoyan@pumch.cn (X.L.); chenmj@pumch.cn (M.C.); zhaojing6503@pumch.cn (J.Z.); zhongwei@pumch.cn (W.Z.); 2 Biomedical Engineering Facility of National Infrastructures for Translational Medicine, Institute of Clinical Medicine, Peking Union Medical College Hospital, Chinese Academy of Medical Sciences and Peking Union Medical College, Beijing 100730, China; lishengjie@pumch.cn; 3 Department of Respiratory and Critical Care Medicine, Baoding No.1 Hospital, Baoding 071000, China; yb2023196@163.com

**Keywords:** lung cancer, young, clinical features, prognosis, immunotherapy, targeted therapy

## Abstract

Background: This study was aimed to comprehensively investigate the clinical and molecular characteristics, treatments, and outcomes of young patients with lung cancer in the new era of cancer treatment. Methods: Clinical data from patients aged 18 to 45 with lung cancer, treated at our hospital from January 2014 through January 2024, were systematically collected and analyzed. Results: This study enrolled a total of 343 patients, with a predominance of females, never-smokers, and those diagnosed at an advanced stage. Adenocarcinoma was the most common histology (72.0%), and rare tumors could also be seen in young patients, such as pulmonary sarcomatoid carcinoma and pulmonary mucoepidermoid carcinoma. The mutation rate of the epidermal growth factor receptor (EGFR) and anaplastic lymphoma kinase (ALK) in NSCLC patients were 35.9% (111/309) and 14.2% (44/309), respectively. PD-L1 expression was assessed in 55 patients, with 14 showing high expression (≥50%) and 24 showing negative expression (<1%). The median overall survival (mOS) for the entire cohort was 80.2 months, with a 5-year survival rate of 55.7%. For patients with stage I, II, and III disease, the mOS had not yet been reached, whereas the mOS for stage IV patients was 39.7 months. Targeted therapy, particularly second-generation ALK tyrosine kinase inhibitors (TKIs), significantly improved the prognosis of patients with driver gene mutations. Chemotherapy combined with immunotherapy was beneficial for patients with progressive disease or driver gene negativity in NSCLC and was associated with improved OS in small cell lung cancer (SCLC). Female, family history of lung cancer, positive driver genes, and first-line use of second-generation ALK-TKIs are independent prognostic factors in young patients with advanced NSCLC. Conclusions: Our findings highlight the importance of early diagnosis, targeted therapy, and immunotherapy in improving outcomes for young patients with lung cancer.

## Simple Summary

Lung cancer in young patients (aged ≤ 45 years) is rare but increasingly recognized as a distinct disease. This study analyzed 343 young lung cancer patients treated between 2014–2024. We found most were female non-smokers with advanced-stage adenocarcinoma. The mutation rates of the EGFR and ALK in NSCLC patients were 35.9% (111/309) and 14.2% (44/309), respectively. Targeted therapies, especially second-generation ALK inhibitors, significantly improved survival. Immunotherapy combined with chemotherapy also benefited patients without driver mutations. The median overall survival was 80.2 months, with a 5-year survival rate of 55.7% for the entire cohort. Our results highlight that early molecular testing and personalized treatments (targeted/immunotherapy) are critical for better outcomes in young lung cancer patients.

## 1. Introduction

The morbidity and mortality of lung cancer has remained the highest globally, exhibiting a disturbing trend [1,2]. Young patients under 45 years old account for a low proportion of lung cancer patients, ranging approximately from about 1% to 3% [3], while in China this value is as high as 6.83% [4,5]. According to cancer statistics in 2016, it is estimated that about 13,000 young adults die of lung cancer in the United States every year [6]. Despite advancements in lung cancer research, the clinical, molecular features and prognosis of young patients remain poorly defined. The rarity of this demographic often leads to their inclusion in studies dominated by elderly patients, potentially obscuring the unique aspects of their disease.

A significant proportion of young patients with lung cancer are non-smokers, with a preponderance of females, suggesting the involvement of alternative etiologies such as genetic predisposition, environmental exposures, and occupational factors [7,8,9,10]. The genomic landscape of lung cancer in young individuals has garnered considerable attention. Although epidermal growth factor receptor (EGFR) mutations and anaplastic lymphoma kinase (ALK) rearrangements are more common in this demographic, the efficacy of targeted therapies in young patients remains controversial [11,12,13,14,15]. Some argue that young patients, with their higher frequency of driver gene mutations, should benefit more from targeted therapy, but clinical research results do not seem to fully support this view [16]. The tumor immune microenvironment plays a crucial role in tumor progression and treatment response [17]. However, it is not fully understood whether there are differences in the tumor immune microenvironment of young patients compared to older patients, such as the composition of tumor-infiltrating lymphocytes and the expression levels of immune checkpoint molecules [18,19]. Meanwhile, the efficacy of immunotherapy in young patients with lung cancer has not been well-elucidated [20].

Existing studies on the prognosis of young lung cancer patients are contentious. Some research suggests that young patients have better prognoses under similar economic conditions, baseline health status, and disease staging [7,12,21,22], while other studies argue that the insidious onset in young patients, often presenting with locally advanced or metastatic disease and poorly differentiated tumor cells, leads to a poorer overall prognosis [8,11,13]. With the emergence of targeted therapies such as the third-generation EGFR-TKIs and the second-generation ALK-TKIs, as well as the rise of immunotherapy, the treatment pattern of lung cancer has undergone revolutionary changes. However, it is not clear whether these advances have particularly improved the prognosis of young patients with lung cancer.

In this study, we retrospectively analyzed clinical/molecular characteristics, treatments, and outcomes in lung cancer patients aged ≤ 45 years to provide new insights into their diagnosis, personalized treatment, and prognosis evaluation in the new era of cancer treatment.

## 2. Patients and Methods

### 2.1. Study Design and Patients

We searched the electronic medical records system in Peking Union Medical College Hospital to identify all relevant records from January 2014 to January 2024. Our search strategy was restricted to the diagnosis of patients including ‘lung cancer’, ‘lung malignancies’, ‘lung carcinomas’, ‘non-small cell lung cancer’, ‘NSCLC’, ‘small cell lung cancer’ or ‘SCLC’, and ‘age ≤ 45 years’. Exclusion criteria: not confirmed by pathology or cytology; mediastinal tumors; non-epithelial-origin tumors such as soft tissue tumors and lymphomas; non-primary lung tumors; patients combined with other types of malignant diseases. Finally, a total of 343 patients aged less than or equal to 45 years old were included.

All study procedures were conducted in accordance with the ethical standards outlined in the 1964 Declaration of Helsinki and its subsequent amendments. The study was approved by the Ethics Review Committee of Peking Union Medical College Hospital, Chinese Academy of Medical Sciences (JS-1410). The informed consent was waived because of retrospective analysis.

### 2.2. Data Collection

Data were extracted through the electronic medical record, including age, gender, family history, smoking history, tumor histology, tumor-node-metastasis (TNM) staging (the 8th American Joint Committee on Cancer (AJCC)) [23], assessment of gene mutations, and treatment. Gene molecular detection mainly used immunohistochemical analysis, fluorescence in situ hybridization (FISH), polymerase chain reaction (PCR), or next-generation sequencing (NGS). Positive driver genes are defined as positive driver genes that are amenable to targeted therapy, including EGFR mutations, ALK rearrangements, ROS-1 rearrangements, RET rearrangements, MET amplifications, or MET exon 14 skipping, among others. Since there were no specific targeted therapies available for KRAS mutations at the time of the study, patients with KRAS mutations were not included in the driver gene positive group. Patients who were EGFR mutation-negative, ALK rearrangement-negative, and had no clearly positive driver gene were defined as the no detectable driver mutations group. Patients with unknown driver gene status were included in the driver gene status unknown group. The expression of programmed death-ligand 1 (PD-L1) was detected by immunohistochemistry using 22C3 antibody kit.

All patients were followed up with through telephone, outpatient, or inpatient visits. The median follow-up duration was 51.4 months (95% confidence interval [CI]: 42.9–59.8 months). For the advanced lung cancer during anti-cancer treatment, the measurable lesions were evaluated by CT/MRI every 6–8 weeks (±1 week), and the efficacy was determined according to the Response Evaluation Criteria in Solid Tumors criteria (RECIST 1.1) until the disease progressed or the treatment was changed [24]. For patients in stage I–III who underwent surgery, thoracoabdominal CT was undergone every 3–6 months to evaluate for disease recurrence. Overall survival (OS) was calculated from the start of treatment until death from any cause. Progression-free survival (PFS) was measured from the start of treatment until the first observation of disease progression or death from any cause. Disease-free survival (DFS) was defined as the time from surgical resection to local recurrence.

### 2.3. Statistical Analysis

Quantitative variables that approximately follow a normal distribution were expressed as means and standard deviations; those with a non-normal distribution were presented as medians and ranges. Qualitative variables were reported as frequencies and percentages. Univariate survival analyses were performed using the Kaplan–Meier method and log-rank test, and survival curves were plotted. The Cox proportional hazards regression was selected for multivariate survival analysis due to its capacity to handle time-to-event data while adjusting for covariates. Statistical analyses in this study were conducted using SPSS 26.0 (SPSS Inc., Chicago, IL, USA) and the R program (version 4.2.0).

## 3. Results

### 3.1. Patient Characteristics

This study enrolled 343 young lung cancer patients, predominantly aged 36–45 years. Non-small cell lung cancer (NSCLC) accounted for 90.1% (*n* = 309) of cases and small cell lung cancer (SCLC) for 9.9% (*n* = 34). A slight female predominance was observed overall (53.6%), which was more marked in the NSCLC group (55.7%) than in the SCLC group (35.3%; *p* = 0.019). The majority of patients were never-smokers (73.8%); however, the proportion of smokers was significantly higher in the SCLC group than in the NSCLC group (55.9% vs. 23.0%, *p* = 0.001). Adenocarcinoma (72.0%) was the most frequent histology, followed by SCLC (9.9%), squamous cell carcinoma (7.9%), and lung carcinoid tumors (6.4%). Rare subtypes such as pulmonary sarcomatoid carcinoma and mucoepidermoid carcinoma were also identified. Over half of the patients (53.9%) had stage IV disease at diagnosis. Among 117 patients who underwent PET/CT, the average maximum standardized uptake value (SUVmax) was 9.3. No significant differences were found between NSCLC and SCLC groups in age, family history, ECOG, or SUVmax (Table 1).

### 3.2. Molecular Features

**Figure 1:**
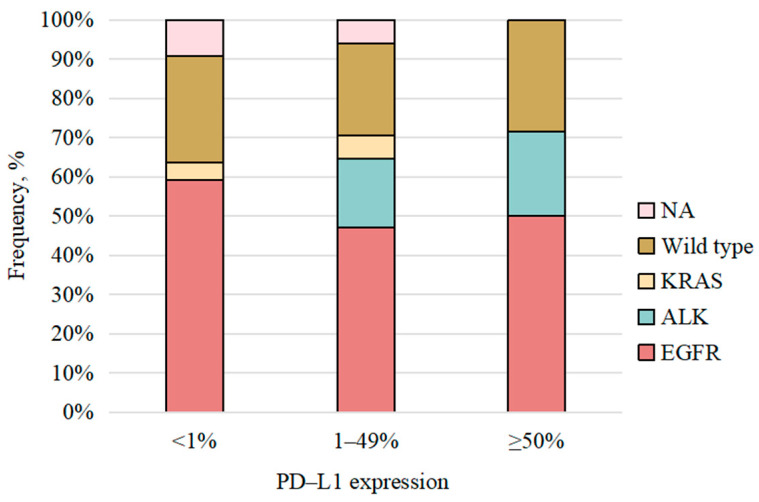
Distribution of gene status frequencies across different levels of PD-L1 expression. PD-L1: programmed death-ligand 1. NA: not applicable.

Of the 34 SCLC patients, none underwent genetic testing; thus, molecular analysis was confined to the 309 NSCLC cases. Actionable driver mutations were found in 52.1% (161/309) of NSCLC patients, being significantly more frequent in adenocarcinoma than non-adenocarcinoma (61.5% vs. 14.5%; *p* < 0.001). EGFR mutations were most common (35.9%), followed by ALK rearrangements (14.2%), especially in adenocarcinoma group (*p* < 0.001). No driver mutations were detected in 18.8% (58/309) of NSCLCs, with no difference between subgroups (*p* = 0.849). Notably, 29.1% (90/309) had unknown driver status due to untested or inadequate samples. Among other alterations, TP53 mutations (8.1%) were most frequent. PD-L1 expression was assessed in 55 patients, with 14 showing high expression (≥50%); most (82.2%) were untested. No significant difference in PD-L1 expression levels was observed between the adenocarcinoma and non-adenocarcinoma groups. Among the 22 patients with available data, the median tumor mutational burden (tTMB) was low, at 2.19 Mut/Mb (Table 2). Figure 1 showed that there was no significant correlation between PD-L1 expression levels and driver gene mutations in this study.

### 3.3. Outcomes of the Overall Population

The median OS for the entire cohort was 80.2 months (95% CI: 62.7–101.7 months). The 1-year, 3-year, and 5-year survival rates of young patients were 94.3%, 71.8%, and 55.7%, respectively. The mOS of patients with stage I, II, and III was not yet reached, while the mOS time of stage IV was 39.7 months (95% CI: 32.7–46.6 months).

### 3.4. Treatment Modalities and Outcomes in NSCLC Patients

A total of 128 NSCLC patients in stages I–III underwent surgery. Among them, 68 of the surgical cases received adjuvant therapy, while eight patients received neoadjuvant therapy. Forty (40/128, 31.3%) had recurrence during follow-up. The median DFS was 18.3 months (95% CI: 13.9–22.6 months). Seventy-six patients received radical radiotherapy for primary and metastatic tumors. Targeted therapy was the most common first-line treatment for advanced or postoperative recurrent patients, followed by chemotherapy, chemotherapy combined with immune checkpoint inhibitors (C + ICIs), and chemotherapy combined with anti-angiogenic monoclonal antibodies (C + Beva), with three patients opting for best supportive care (Table 3).

**Figure 2:**
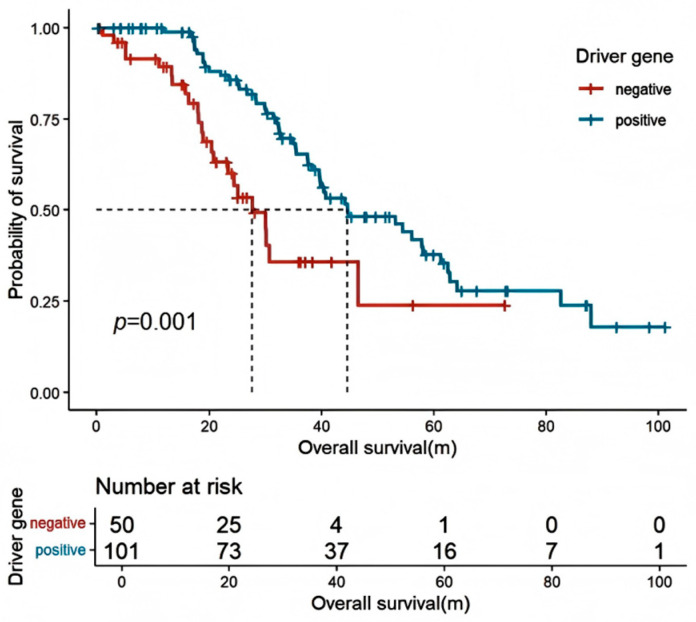
Correlation between OS and driver gene mutations in all locally advanced/advanced NSCLC patients who underwent driver gene testing. OS: overall survival.

**Figure 3:**
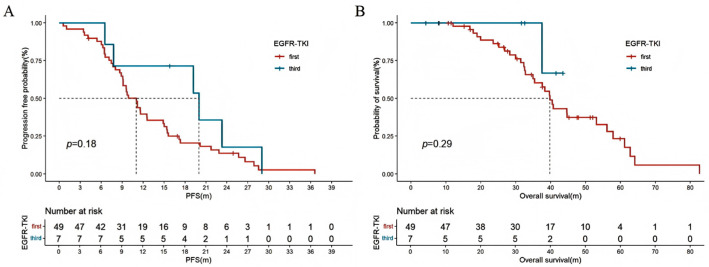
Kaplan–Meier estimates of PFS (**A**) and OS (**B**) in the first- and third-generation of EGFR-TKIs. PFS: progression-free survival; OS: overall survival. EGFR-TKIs: epidermal growth factor receptor-tyrosine kinase inhibitors.

**Figure 4:**
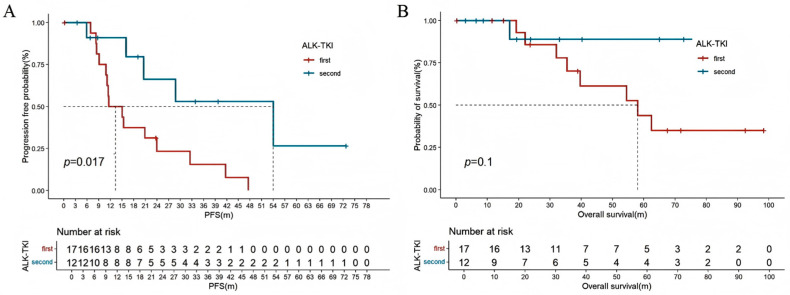
Kaplan–Meier estimates of PFS (**A**) and OS (**B**) in the first- and second-generation of ALK-TKIs. PFS: progression-free survival; OS: overall survival. ALK-TKIs: anaplastic lymphoma kinase-tyrosine kinase inhibitors.

Among all patients with locally advanced or metastatic NSCLC who underwent driver gene testing (*n* = 151), patients with positive driver gene mutations had a longer OS compared to those with no detectable driver mutations (44.6 [95% CI: 30.6–58.6] vs. 32.8 [95% CI: 10.7–54.9] months, HR: 0.636 [95% CI: 0.389–0.892], *p* = 0.001) (Figure 2). In advanced lung cancer patients with EGFR-positive mutations, there was no significant difference in PFS (11.0 [95% CI: 9.2–12.8] vs. 20.0 [95% CI: 2.4–37.6] months, HR: 1.560 [95% CI: 0.656–3.708], *p* = 0.18) and OS (39.8 [95% CI: 36.3–43.2] months vs. not reached, HR: 2.827 [95% CI: 0.381–20.991], *p* = 0.29) between first-line use of first-generation or third-generation EGFR-tyrosine kinase inhibitors (EGFR-TKIs) (Figure 3A,B). For patients harboring ALK-sensitive mutations, first-line use of first-generation ALK-TKIs resulted in shorter PFS compared to second-generation ALK-TKIs (11.5 [95% CI: 4.5–18.6] vs. 28.8 [95% CI: 16.4–61.0] months, HR: 3.122 [95% CI: 1.023–9.529], *p* = 0.017). Although there was no statistical difference in OS, the separation in the curves was noticeable (58.1 [95% CI: 28.6–87.6] vs. not reached, HR: 4.844 [95% CI: 0.605–38.806], *p* = 0.1) (Figure 4A,B).

**Figure 5:**
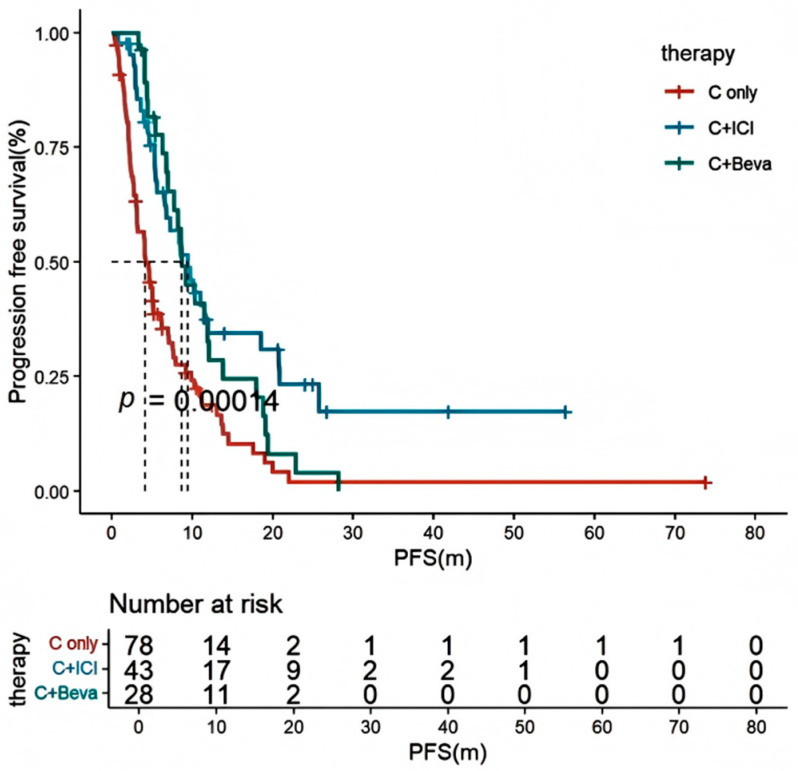
Kaplan–Meier estimates of PFS in different treatment modalities for patients with targeted therapy progression or no detectable driver mutations. Of these, C + ICI significantly prolonged PFS compared to chemotherapy alone (*p* = 0.0098), whereas C + Beva did not show statistical difference compared to chemotherapy alone (*p* = 0.0516). PFS: progression-free survival; C: chemotherapy; C + ICI: chemotherapy combined with immune checkpoint inhibitors; C + Beva: chemotherapy combined with anti-angiogenic monoclonal antibodies Bevacizumab.

In young patients who had progressed on targeted therapy or were not detectable for driver genes, the PFS was significantly longer with the immuno-chemotherapy compared to chemotherapy alone (9.4 [95% CI: 6.1–12.8] vs. 4.2 [95% CI: 3.3–5.1] months, HR = 0.575 [95% CI: 0.247–0.857], *p* < 0.001). There was no statistical difference between the chemotherapy and anti-angiogenic therapy versus chemotherapy alone (*p* > 0.05) (Figure 5).

Among young patients with advanced NSCLC, women (HR: 0.556, 95% CI: 0.355–0.870), positive driver gene mutation (HR: 0.590, 95% CI: 0.373–0.935), and first-line second-generation ALK-TKIs (HR: 0.181, 95% CI: 0.025–0.317) were associated with improved prognosis, while family history of lung cancer (HR: 2.643, 95% CI: 1.543–4.528) was a risk factor (Table 4).

### 3.5. Outcomes in Young Patients with SCLC

Among the 34 young patients with SCLC, two cases were in stage I and underwent curative surgery, with no recurrence observed, demonstrating a DFS of 59.63 and 9.6 months, respectively. Fourteen patients were in stage III, with 13 receiving concurrent chemoradiotherapy and one receiving immuno-chemotherapy. The mOS for this group was 35.7 months (95% CI: 12.5–73.1), and the mPFS was 17.3 months (95% CI: 6.8–26.3). Eighteen patients were in stage IV, of which 11 received chemotherapy alone and seven received immuno-chemotherapy. There was no significant difference in PFS between the two groups. However, the group treated with immuno-chemotherapy exhibited a markedly prolonged OS compared to the chemotherapy group (31.5 [95% CI: 16.2–46.7] vs. 14.8 [95% CI: 5.3–24.2] months, HR: 0.130 [95% CI: 0.016–0.370], *p* = 0.028).

## 4. Discussion

This study retrospectively analyzed the clinicopathological features, treatment, and prognosis of young lung cancer patients, and revealed the therapeutic response of young lung cancer to targeted therapy and immunotherapy, highlighting the heterogeneity and uniqueness of this special population in lung cancer treatment.

The pathogenesis of young-onset lung cancer involves specific driver mutations and a unique immune microenvironment [25]. Mutations in genes such as EGFR, ALK, and ROS1 are prevalent in younger patients, promoting cell proliferation and tumor growth, while mutations in tumor suppressor genes like BRCA2 and TP53 increase lung cancer susceptibility, particularly in non-smoking individuals [9,11,12]. Additionally, young-onset lung cancers often exhibit lower TMB, which, along with an increase in immunosuppressive cells, weakens immune surveillance, allowing tumors to evade immune detection more effectively [19]. Together, these genetic and immune characteristics form a distinctive pathological profile in young-onset lung cancer.

Our findings are consistent with the existing literature, indicating a higher proportion of female and non-smoking young lung cancer patients, with adenocarcinoma being the main pathological type and some patients reporting a family history of lung cancer [7,8,9,10]. Previous studies have reported EGFR mutation rates in young lung cancer ranging from 32% to 46.9%, and ALK rearrangement rates from 6.3% to 19%, which are consistent with those reported in this study [11,12,13,14,15]. Adrian G. et al.’s research also supports this, pointing out that EGFR and ALK gene mutations are associated with a younger age at cancer diagnosis [11]. The high incidence of driver gene mutations in young patients also suggests potential therapeutic advantages. Squamous cell carcinoma is rarely sequenced due to current lack of targetable therapies, though gene mutations may exist. Targeting the molecular and immune microenvironment is crucial for understanding squamous cell carcinoma in young patients. It is noteworthy that our cohort exclusively comprised Chinese patients, among whom EGFR mutations are significantly more prevalent compared to Caucasian (10–15%) and African-American populations (5–10%) [11,12,13,14,15]. This limits the generalizability of our findings to non-Asian cohorts. Future multi-ethnic studies are warranted to validate these results globally.

A high proportion and response rate were observed in patients carrying driver gene mutations who received targeted therapy as the first line, highlighting the importance of personalized medicine in this population. Both randomized controlled clinical trials and real-word studies of third-generation EGFR TKIs significantly prolonged PFS and OS in patients with advanced EGFR-mutated NSCLC compared to first-generation EGFR-TKIs [26,27]. This study suggests that first-line use of third-generation EGFR-TKIs may be also beneficial for young patients. The lack of statistical significance may be related to the small number of patients receiving third-generation EGFR-TKIs due to the large treatment time span included in this study, resulting in a smaller number of patients using third-generation EGFR-TKIs as the first line. The ALEX study demonstrated that first-line alectinib had significant advantages over crizotinib in PFS and OS [28]. A network meta-analysis showed that alectinib is proven to be the first choice for first-line and second-line treatment of advanced lung cancer with ALK mutations [29]. This study also indicates that, compared to first-generation TKIs, second-generation ALK-TKIs significantly prolong PFS in young patients. It is noteworthy that the PFS with second-generation ALK-TKIs in the ALEX study is notably longer than the PFS observed in young patients in this study (34.8 months vs. 28.8 months). This is likely due to differences in the age distribution of the included populations and varying patterns of concurrent mutations among patients. In Tian et al.’s study, young patients with ALK fusions had a significantly different PFS compared to older patients who were treated with first-line ALK-TKIs (17.5 months vs. 9.0 months, *p* = 0.048) [16]. However, young patients with concurrent TP53 mutations had a median PFS of only 6.2 months [16]. Current research on the efficacy of targeted therapy in young patients with lung cancer is relatively limited, and the impact of age on the efficacy of targeted therapy has not been fully elucidated. More large-scale, prospective studies, as well as basic research, are needed to further explore this relationship.

Immunotherapy may play a pivotal role in the treatment of young patients with lung cancer, particularly in those with targeted therapy progression or driver gene negativity. Studies have shown that immuno-chemotherapy can significantly improve PFS in patients with advanced EGFR-mutated tumors compared to chemotherapy alone (6.7 months vs. 4.2 months, *p* = 0.0076) [30]. However, a multicenter, retrospective study showed that patients under 55 years old who received ICIs had an increase in median survival of 4 to 5 months, while no significant survival improvement was observed in patients over 75 years old [31]. Young patients with advanced NSCLC who received immuno-chemotherapy in this study appeared to have better PFS than older patients (9.4 months vs. 6.7 months), which may be related to differences in the tumor microenvironment. Notably, this study found that only a few patients had high expression. The tumor tissue also showed low TMB characteristics. This is consistent with the research reported by Rossana Ruiz et al. [15]. Luo et al. found that tumors in non-smoking patients express lower levels of PD-L1 and lower TMB [25]. Researchers have shown that aging impairs the persistence and proliferation of antigen-specific CD8+ T cells, while reducing the infiltration of NK cells, cDC1 cells, and other cells in the tumor microenvironment, affecting the interactions between immune cells and ultimately weakening the anti-tumor immune response [18,19]. Yajas et al. investigated differences in tumor characteristics related to age across a broad spectrum of cancers and found that compared to tumors from elderly patients, those from younger patients contained a higher number of infiltrating immune cells, indicating that young patients can mount a stronger immune response to more lethal cancers [32]. It was also discovered that the mechanistic target of the pembrolizumab may serve as a promising candidate for targeted therapy in uterine cancer among young patients [32]. These findings may be one of the reasons why young patients receive immunotherapy with better efficacy than older patients. However, other findings indicate that with increasing age, there is an increase in immune-related biomarkers, and the elderly group may have a better response to immunotherapy compared to the younger group [20], which suggests that the tumor immune microenvironment in young individuals may have unique and complex characteristics. However, it should be noted that the detection rate of PD-L1/tTMB in this population is very low, which is mainly due to the following factors: a. the limitations of the period, from earlier years when testing was not routine; b. clinical prioritization of detecting actionable driver mutations (e.g., EGFR, ALK) for guiding first-line therapy, particularly in adenocarcinoma; c. limited tissue availability. Future research should focus on revealing the features and differences of the tumor immune microenvironment in young lung cancer patients and how to optimize immunotherapy strategies to improve treatment outcomes in this specific population.

The controversy surrounding the prognosis of young lung cancer patients reflects the complexity of disease treatment in this population. Brian N. et al. found that young NSCLC patients have better overall and relative survival rates than older patients, with greater benefits for stage I and II patients. Compared with older patients, active multimodal treatment, including surgery, is more feasible and effective for young patients [12]. The survival analysis of our cohort, with a median follow-up time of 51.4 months, shows a promising OS of 80.2 months, which may be related to early tumor detection, high treatment willingness, the widespread application of targeted and immunotherapy, and better economic conditions. This also reveals the importance of early diagnosis and intervention in this young population.

We must acknowledge the limitations of this study, including the single-center retrospective design, relatively small sample size, inherent selection bias (e.g., tertiary-center referrals), and the lack of some clinical data. These limitations may affect the generalizability of our findings. Future prospective studies should further expand the sample size, focusing on and exploring age-specific immunotherapy biomarkers and risk-based screening models for young non-smokers, and explore the multi-omics characteristics of tumor immune microenvironment aging [33].

## 5. Conclusions

This study reveals the unique clinical characteristics and outcomes of lung cancer in young Asian patients, emphasizing the importance of early diagnosis and intervention in this young population. The high rate of driver gene mutations and the potential benefits of combined therapy highlight the necessity for further research into the molecular feature and tumor microenvironment of lung cancer in young patients.

## Figures and Tables

**Table 1:** Baseline demographic and clinical characteristics in the overall population.

Variable	Overall Population (N = 343)	NSCLC Group (N = 309)	SCLC Group (N = 34)	*p* *
Sex				0.019
Men	159 (46.4)	137 (44.3)	22 (64.7)	
Women	184 (53.6)	172 (55.7)	12 (35.3)	
Age, years				0.896
36–45	259 (75.5)	232 (75.1)	27 (79.4)	
26–35	63 (18.4)	58 (18.8)	5 (14.7)	
≤25	21 (6.1)	19 (6.1)	2 (5.9)	
Smoking history				0.001
Never smokers	253 (73.8)	238 (77)	15 (44.1)	
Current/former smokers	90 (26.2)	71 (23)	19 (55.9)	
Smoking index (pack year)	13.5 ± 6.2	12.0 ± 5.7	20.4 ± 11.2	0.010
Family history of cancer	95 (27.7)	86 (27.8)	9 (26.5)	0.523
Family history of lung cancer	52 (15.2)	45 (14.6)	6 (17.6)	0.393
ECOG				0.488
0–1	317 (92.4)	286 (92.6)	31 (91.2)	
≥2	26 (7.6)	23 (7.4)	3 (8.8)	
Type				0.001
Central lung cancer	109 (31.8)	86 (27.8)	30 (88.2)	
Peripheral lung cancer	234 (68.2)	223 (72.2)	4 (11.8)	
Histology				/
NSCLC	309 (90.1)	309	/	
Adenocarcinoma	247 (72.0)	247 (79.9)		
Squamous cell carcinoma	27 (7.9)	27 (8.7)		
Lung carcinoid	22 (6.4)	22 (7.1)		
Adenosquamous carcinoma	7 (2.0)	7 (2.3)		
Large cell carcinoma	2 (0.6)	2 (0.6)		
NOS	2 (0.6)	2 (0.6)		
Pulmonary sarcomatoid carcinoma	1 (0.3)	1 (0.3)		
Pulmonary mucoepidermoid carcinoma	1 (0.3)	1 (0.3)		
SCLC	34 (9.9)	/	34	
Staging				0.001
I	72 (21.0)	70 (22.7)	2 (5.9)	
II	22 (6.4)	22 (7.1)	0 (0)	
III	64 (18.7)	50 (16.2)	14 (41.2)	
IV	185 (53.9)	167 (54)	18 (52.9)	
Distant metastasis				0.697
Pleural	97 (52.4)	90	7	
Bone	91 (49.2)	85	6	
Intrapulmonary	75 (40.5)	69	6	
Brain	46 (24.9)	42	4	
Pericardium	34 (18.4)	33	1	
Liver	24 (13.0)	18	6	
Adrenal gland	17 (9.2)	14	2	
Leptomeningeal	3 (1.6)	3	0	
Others ^#^	14 (7.6)	13	1	
SUVmax (*n* = 117)	9.3 ± 6.3	9.2 ± 6.4	10.8 ± 5.1	0.338

Values are mean ± SD, *n* (%). * NSCLC group vs. SCLC group. ^#^ Including skin, abdominal, and pelvic lymph nodes, axillary lymph nodes, muscle, pancreas, kidney, and other rare metastatic sites. Abbreviations: ECOG: Eastern Cooperative Oncology Group; NOS: not otherwise specified; SUV: standardized uptake value; TMB: tumor mutation burden.

**Table 2:** The molecular characteristics of young patients with NSCLC.

Molecular Characteristics	Overall NSCLC (N = 309)	Adenocarcinoma (N = 247)	Non-Adenocarcinoma (N = 62)	*p* *
Driver gene positive	161 (52.1)	152 (61.5)	9 (14.5)	<0.001
EGFR mutation	111 (35.9)	105 (42.5)	6 (9.7)	<0.001
exon 19 del	64 (20.7)	61 (24.7)	3 (4.8)	
exon 21 L858R	28 (9.1)	26 (10.5)	2 (3.2)	
exon 20 ins	14 (4.5)	13 (5.3)	1 (1.6)	
exon 18 G719X	2 (0.6)	2 (0.8)	0	
exon 21 L861Q	1 (0.3)	1 (0.4)	0	
exon 18 E709X	1 (0.3)	1 (0.4)	0	
exon 18 G719A	1 (0.3)	1 (0.4)	0	
ALK rearrangement	44 (14.2)	41 (16.6)	3 (4.8)	<0.001
EML4-ALK fusion	26 (8.4)	23 (9.3)	3 (4.8)	
HIP1-ALK fusion	1 (0.3)	1 (0.4)	0	
KLC1-ALK fusion	1 (0.3)	1 (0.4)	0	
Unknown	16 (5.2)	16 (6.4)	0	
ROS1 rearrangement	6 (1.9)	6 (2.4)	0	0.612
c-MET exon14 skipping or c-MET amplification	3 (1.0)	3 (1.2)	0	0.438
RET rearrangement	3 (1.0)	3 (1.2)	0	0.716
No detectable driver mutations	58 (18.8)	47 (19.0)	11 (17.7)	0.849
Driver gene unknown	90 (29.1)	48 (19.4)	42 (67.7)	<0.001
Other gene mutations detected				/
TP53	25 (8.1)	22 (8.9)	3 (4.8)	
HER-2	7 (2.3)	6 (2.4)	1 (1.6)	
KRAS G12D	3 (1.0)	3 (1.2)	0	
BRCA	2 (0.6)	2 (0.8)	0	
MET exon21skipping	2 (0.6)	2 (0.8)	0	
MET protein overexpression	1 (0.3)	1 (0.4)	0	
MET gene fusion	1 (0.3)	1 (0.4)	0	
BRAF-V600E	1 (0.3)	1 (0.4)	0	
NTRK1	1 (0.3)	0	1 (1.6)	
PD-L1 expression				0.480
<1%	24 (7.8)	21 (8.5)	3 (4.8)	
1–49%	17 (5.5)	14 (5.7)	3 (4.8)	
≥50%	14 (4.5)	13 (5.3)	1 (1.6)	
Unknown	254 (82.2)	199 (80.6)	55 (88.7)	
tTMB(Muts/Mb), median (Q1–Q3)	22 (7.1)	2.19 (1.60, 4.82)	NA	/

* Adenocarcinoma group vs. Non-adenocarcinoma group. Abbreviations: NA: not applicable; Q: quartile.

**Table 3:** Treatments in the NSCLC patients, N = 309.

Therapy	*n* (%)
Surgery	128 (41.4)
Adjuvant therapy	68 (53.1)
Neoadjuvant therapy	8 (6.3)
Postoperative recurrence	40 (31.3)
Radical radiotherapy	76 (24.6)
First line therapies in advanced stage	
Target therapy	116 (37.5)
C only	58 (18.8)
C + ICIs	24 (7.8)
C + Beva	22 (7.1)
Best supportive care	3 (1.0)

Abbreviations: C: chemotherapy; C + Beva: chemotherapy combined with anti-angiogenic monoclonal antibodies Bevacizumab; C + ICIs: chemotherapy combined with immune checkpoint inhibitors.

**Table 4:** Cox regression prognostic variables for OS in young patients with advanced NSCLC.

Variable	Univariate Analyses		Multivariate Analyses	
	HR (95% CI)	*p* Value	HR (95% CI)	*p* Value
Sex				
men	re		re	
women	0.562 (0.359–0.880)	0.012	0.556 (0.355–0.870)	0.010
Family history of lung cancer				
No	re		re	
Yes	2.240 (1.424–4.114)	0.001	2.643 (1.543–4.528)	0.001
Smoking history				
Never smokers	re			
Current/former smokers	1.406 (0.836–2.366)	0.199		
ECOG				
0–1	re			
≥2	1.063 (0.562–2.008)	0.852		
Histology				
squamous cell carcinoma	re			
non-squamous NSCLC	0.918 (0.288–2.933)	0.886		
PD-L1 expression				
Negative	re			
positive	0.866 (0.206–3.634)	0.844		
NA	1.526 (0.478–4.868)	0.475		
Driver gene mutation				
No detectable	re		re	
positive	0.564 (0.361–0.881)	0.012	0.590 (0.373–0.935)	0.025
NA	0.566 (0.305–1.052)	0.072		
EGFR-TKI				
no	re			
First	1.149 (0.729–1.812)	0.550		
Third	0.390 (0.054–2.837)	0.352		
ALK-TKI				
no	re		re	
First	0.499 (0.239–1.042)	0.064		
Second	0.132 (0.018–0.954)	0.045	0.181 (0.025–0.317)	0.041
Immunotherapy				
no	re			
yes	1.106 (0.478–2.562)	0.813		
Radical radiotherapy				
no	re			
yes	0.791 (0.510–1.226)	0.294		

Abbreviations: ECOG: Eastern Cooperative Oncology Group; PD-L1: programmed death-ligand 1; EGFR: epidermal growth factor receptor; ALK: anaplastic lymphoma kinase; re: reference.

## Data Availability

The data presented in this study are available on request from the corresponding authors.
